# Hepatic responses as potential biomarkers of idiosyncratic drug reaction risk

**DOI:** 10.1093/toxsci/kfag087

**Published:** 2026-07-29

**Authors:** Jiamin Liao, Xiaotian Lyu, Kristy Yang, Lucia Chen, Jack Uetrecht

**Affiliations:** Department of Pharmaceutical Sciences, University of Toronto, Toronto, ON M5S 3M2, Canada; Department of Pharmacology and Toxicology, University of Toronto, Toronto, ON M5S 3M2, Canada; Department of Pharmaceutical Sciences, University of Toronto, Toronto, ON M5S 3M2, Canada; Department of Pharmacology and Toxicology, University of Toronto, Toronto, ON M5S 3M2, Canada; Department of Pharmaceutical Sciences, University of Toronto, Toronto, ON M5S 3M2, Canada; Department of Pharmacology and Toxicology, University of Toronto, Toronto, ON M5S 3M2, Canada; Department of Pharmaceutical Sciences, University of Toronto, Toronto, ON M5S 3M2, Canada; Department of Pharmacology and Toxicology, University of Toronto, Toronto, ON M5S 3M2, Canada; Department of Pharmaceutical Sciences, University of Toronto, Toronto, ON M5S 3M2, Canada; Department of Pharmacology and Toxicology, University of Toronto, Toronto, ON M5S 3M2, Canada

**Keywords:** idiosyncratic drug reactions, idiosyncratic drug-induced liver injury, risk biomarkers, innate immune response

## Abstract

Idiosyncratic drug reactions (IDRs) and, in particular, idiosyncratic drug-induced liver injury (iDILI) represent a significant risk for drug development. There are multiple lines of evidence that such reactions are mediated by the adaptive immune system. The idiosyncratic nature of adaptive immune responses makes it difficult to develop biomarkers to predict the risk that drug candidates will cause such adverse reactions. However, an adaptive immune response requires an innate immune response to activate antigen-presenting cells such as macrophages, and the innate immune response to drugs should not be idiosyncratic. We had previously shown that clozapine and nevirapine produce a marked innate immune response in rodents. We sought to extend these studies to additional drugs that cause IDRs; namely, amodiaquine, carbamazepine, isoniazid, and ximelagatran. Ximelagatran was specifically chosen because, unlike the others, it does not appear to form a reactive metabolite. Amodiaquine and carbamazepine also produced changes associated with an innate immune response, such as an increase in serum corticosterone and GDF-15 and changes in hepatic mRNA *for Dusp1*, *Gadd45b*, and *Cebpd*. These changes resolved within hours. Not surprisingly, ximelagatran did not produce significant changes. However, it was surprising that isoniazid produced few such changes. There is evidence that ximelagatran can directly activate macrophages, and it is plausible that isoniazid can also directly activate macrophages. It is likely that changes in hepatic mRNA consistent with a stress/innate immune response would represents biomarkers of iDILI risk; however, some drugs may directly activate macrophages without the need of factors released from the liver.

Idiosyncratic drug reactions (IDRs), especially idiosyncratic drug-induced liver injury (iDILI) represent a major risk for drug development (Watkins et al. 2011). There is currently no accurate way to predict the risk that a drug will cause such adverse reactions, and they are usually only discovered late in clinical trials or even after the drug has been marketed. It is unlikely that better biomarkers of IDR risk can be developed without a better mechanistic understanding of the mechanisms involved. It is now clear that the majority of IDRs are mediated by the adaptive immune system. Although the immune response is very complex and involves many cell types, the most serious reactions such as toxic epidermal necrolysis and hepatocellular iDILI are mediated principally by CD8+ T cells (Nassif et al. 2004; Foureau et al. 2015). Although there are likely many unknown risk factors, for an individual the most likely risk factors are the requirement for specific human leukocyte antigen molecules (HLA) to present the drug-associated antigen, and specific T cell receptors to recognize these drug-associated antigens (Uetrecht 2025). These factors that make IDRs idiosyncratic make the study of IDRs very difficult. However, to elicit an adaptive immune response, an innate immune response is required to activate antigen-presenting cells (APCs) such as macrophages and dendritic cells. This is referred to as signal 2, which is fundamental to the Danger Hypothesis. This explains why foreign proteins do not usually produce an immune response unless they pose a danger to the organism and why vaccines require an adjuvant (Matzinger 1994). The danger hypothesis has important relevance to idiosyncratic drug reactions because it can explain why not all drugs that form reactive metabolites that modify proteins are associated with a significant risk of IDRs (Uetrecht 2025). Classically, this activation requires the release of damage-associated molecular pattern molecules (DAMPS) that activate APCs through toll-like receptors (Matzinger 2002). This innate immune response does not require specific HLA molecules or TCRs and should not be idiosyncratic. The range of possible mechanisms of APC activation is quite broad, but it provides the best avenue to discover accurate biomarkers of IDR risk.

As a test of the innate immune response hypothesis, we have previously studied the initial response to clozapine and nevirapine; 2 drugs associated with a relatively high incidence of IDRs. We found that treatment of rats with clozapine led to a rapid increase (3 h) in serum CXCL1, serum corticosterone, IL-1β, and GDF-15 (Sernoskie et al. 2023, 2024). There is a later increase in the acute phase protein α1-acid glycoprotein (Sernoskie et al. 2023). Likewise, nevirapine treatment led to an increase in serum corticosterone and GDF-15 (Jee et al. 2024). It also led to an increase in mRNA levels of several molecules involved in the stress/innate immune response such as Gadd45b, Dusp1, CEBPD, and Hspa2. With daily nevirapine treatment, the level of serum corticosterone returned to normal by 3 d suggesting some form of adaptation or tolerance.

We set out to test whether such a response could represent biomarkers of IDR risk. The drugs we chose to study were amodiaquine, carbamazepine, isoniazid, and ximelagatran. The first 3 drugs are associated with a variety of IDRs and are metabolized to reactive metabolites, which are assumed to be related to their ability to cause IDRs (Lu and Uetrecht 2008; Metushi et al. 2012; Lobach and Uetrecht 2014). In contrast, ximelagatran is associated with iDILI, but does not appear to form a reactive metabolite; therefore, it may produce a different type of response (Kenne et al. 2008). In fact, it has been reported that ximelagatran directly activates macrophages (Edling et al. 2008); therefore, activation by signals from the liver may not be required.

## Materials and methods

### Chemicals

All reagents were commercially obtained. Acetaminophen, amodiaquine, carbamazepine, isoniazid, and promethazine hydrochloride were purchased from Sigma-Aldrich (St. Louis, MO, United States). Amodiaquine-d10, ximelagatran, and melagatran were purchased from Cayman Chemical (Ann Arbor, MI, United States).

### Animals

Six- to 8-wk-old female C57BL/6NCrl mice (18 to 25 g) were obtained from Charles River Laboratories (Saint-Constant, QC, Canada) and housed in groups of 4 per cage. Animals were acclimated for at least 1 wk before experimentation under the supervision of staff in the Division of Comparative Medicine, University of Toronto. All procedures were approved by the University of Toronto Animal Care Committee.

### Drug administration

Carbamazepine (100 mg/kg), amodiaquine (200 mg/kg), isoniazid (150 mg/kg), and ximelagatran (95 mg/kg) were administered by intraperitoneal injection and formulated in 20% Captisol (sulfobutylether-β-cyclodextrin; SBECD) in 0.9% saline. The control animals were given an equivalent volume of drug solvent.

In preliminary studies, the drugs were administered by oral gavage. However, there was a large variation in the resulting blood levels of the drug (data not shown). Therefore, drug administration was changed to the ip mode. Doses were selected based on preliminary studies to achieve mean blood concentrations that were modestly above typical therapeutic ranges in humans to minimize artifacts associated with toxicity and reduce false-negative outcomes, as lower exposures may fail to capture higher (“outlier”) concentrations that could be relevant to IDR risk in humans. In addition, therapeutic concentrations usually refer to trough levels rather than Cmax, and the levels that we measured are closer to Cmax. Carbamazepine was the highest concentration. The therapeutic concentration in humans is about 10 µg/ml. All but one of the carbamazepine concentrations was modestly above this concentration. The therapeutic concentration of isoniazid is about 5 µg/ml, but concentrations vary significantly in humans based on acetylator phenotype, which is also a risk factor for isoniazid iDILI (Daly 2017). All of the isoniazid concentrations in our study were modestly above this level. The concentrations of amodiaquine were all above the therapeutic concentration, but amodiaquine is concentrated in the liver, and the liver concentration increases with multiple doses relative to the serum concentration (Winstanley et al. 1988). In this single dose study, the liver concentration would be lower than expected from the serum concentration. However, we used a similar amodiaquine dose in a chronic study in PD-1 KO mice. They developed delayed mild liver injury that was mediated by CD8+ T cells without any other evidence of toxicity (Mak and Uetrecht 2015a). The dose of ximelagatran is based on studies by Lundgren et al. (2017) in which they produced maximal pharmacological effects in mice without toxicity.

### Tissue collection

Mice were euthanized by carbon dioxide asphyxiation followed by cardiac puncture for blood collection. Blood was transferred into serum microtubes containing clot activator (Sarstedt, Montréal, QC, Canada) and centrifuged at 10,000 × g for 1.5 min to obtain serum. The liver and spleen were then dissected, rinsed in PBS, and weighed. Approximately 50 mg of liver tissue was submerged in RNAlater Stabilization Solution (Thermo Fisher Scientific, Waltham, MA, United States), kept on ice, and subsequently stored at −20°C until RNA extraction. All dosing and tissue collection were performed within the same time window to minimize circadian variation in corticosterone.

### Liquid chromatography–tandem mass spectrometry

Serum samples were extracted by protein precipitation with methanol containing the internal standards promethazine hydrochloride, acetaminophen, melagatran, and amodiaquine-d10. Samples were incubated at −20°C for 20 min and centrifuged at 13,200 rpm for 10 min. Supernatants were transferred to LC vials and analyzed on a 1260 Infinity II Prime LC system (Agilent Technologies, Santa Clara, CA, United States) coupled to a TSQ Endura triple-stage quadrupole mass spectrometer (Thermo Fisher Scientific).

Chromatographic separation was performed on an Agilent InfinityLab Poroshell 120 EC-C18 column (Agilent Technologies; 3.0 × 50 mm, 2.7 µm) at a flow rate of 0.3 ml/min with an injection volume of 5 µl using analyte-specific conditions optimized for each compound. Mobile phases consisted of water containing 0.1% formic acid and either acetonitrile or methanol as the organic phase. Quantitation was performed in XCalibur (version 4.0; Thermo Fisher Scientific) using analyte-specific calibration curves prepared in blank mouse serum over a range of 0 to 50 μg/ml. Final concentrations were calculated from the analyte-to-internal standard peak-area ratio.

### Real-time quantitative polymerase chain reaction

Liver tissue (50 mg) was homogenized in 750 µl TRIzol reagent (Thermo Fisher Scientific). Chloroform (100 µl) was added, and samples were vortexed, incubated at room temperature for 5 min, and centrifuged at 13,200 rpm for 15 min. The aqueous phase was mixed 1:1 with freshly prepared 70% ethanol, and total RNA was purified using the Aurum Total RNA Mini Kit (Bio-Rad Laboratories, Hercules, CA, United States) according to the manufacturer’s instructions. RNA concentration and quality were assessed using an Ultrospec 2100 pro UV/Vis spectrophotometer (Biochrom Ltd, Cambridge, England), followed by cDNA synthesis using SuperScript IV Reverse Transcriptase kit, RNaseOUT Recombinant Ribonuclease Inhibitor, dNTP Mix, (Thermo Fisher Scientific).

Quantitative real-time PCR (qPCR) was performed using cDNA corresponding to 12.5 ng RNA and PowerUp SYBR Green Master Mix (Applied Biosystems/Thermo Fisher Scientific). Gene expression was normalized to the housekeeping gene cyclophilin (CyPs). Target genes were selected based on prior studies reporting early hepatic transcriptional changes induced by IDR-associated drugs, particularly nevirapine (Jee et al. 2024) and clozapine (Sernoskie et al. 2022). Primer sequences can be found in Table S1.

Reactions were run on a CFX384 Touch Real-Time PCR Detection System (Bio-Rad) in technical triplicate using the following cycling conditions: 50°C for 2 min, 95°C for 10 min, and 44 cycles of 95°C for 15 s and 60°C for 1 min. Relative fold changes were calculated using Bio-Rad CFX Maestro Software (version 1.1).

### RNA-sequencing

RNA-sequencing (RNA-seq) analyses were performed by The Centre for Applied Genomics at The Hospital for Sick Children (Toronto, Ontario). Total RNA was isolated from liver tissue collected 3 h after treatment from 7- to 9-wk-old mice administered vehicle or carbamazepine/isoniazid. Eight samples were analyzed with 4 biological replicates per group. RNA quality was assessed by the sequencing facility, and libraries were prepared for mRNA sequencing. Samples were submitted for paired-end 100-bp sequencing on an Illumina NovaSeq SP flowcell.

### RNA-seq data analysis

Reads were assessed using FastQC version 0.11.5 and trimmed using Trim Galore version 0.5.0 (Cutadapt version 1.10). Additional quality-control analyses were performed using FastQ Screen version 0.10.0, RSeQC version 2.6.2, and MultiQC version 1.14. Trimmed reads were aligned to the mouse reference genome GRCm39 with GENCODE M35 annotation using STAR aligner version 2.6.0c, and raw gene counts were generated using htseq-count version 0.6.1p2. Differential gene expression analysis was performed in R version 3.6.1 using DESeq2 version 1.26.0, with cage included as a covariate. Genes with fewer than 20 counts in at least 3 samples were excluded prior to analysis.

Exploratory analyses included boxplots of expression distributions and PCA, MDS, and sample clustering based on rlog-transformed counts. Volcano plots and heatmaps were generated from DESeq2 results, and FPKM values for the unfiltered gene set were also calculated.

### Enzyme-linked immunosorbent assay

DuoSet enzyme-linked immunosorbent assay (ELISA) kits (R&D Systems, Minneapolis, MN, United States) were used to quantify GDF-15 in mouse serum. Serum corticosterone was measured using ELISA kits from Invitrogen/Thermo Fisher Scientific. All assays were performed according to the manufacturer’s instructions.

### Statistical analysis

Statistical analyses were performed using GraphPad Prism (version 9.5.1; GraphPad Software, San Diego, CA, United States). For comparisons between 2 independent groups, an unpaired 2-tailed Student’s *t*-test was used. For experiments involving more than 2 groups, 1-way ANOVA was performed, followed by Dunnett’s multiple-comparisons test versus vehicle. For experiments with 2 independent variables, data were analyzed by 2-way ANOVA followed by Šídák’s multiple-comparisons test. Data are presented as mean ± SEM for qPCR and mean ± SD for all other measurements. A *P*-value <0.05 was considered statistically significant.

## Results

### Time-course analysis defines the early kinetics of the amodiaquine-induced response

To define an appropriate time window for detecting early drug-induced responses in vivo, a focused time-course study was first performed using amodiaquine. Mice were sampled at 2, 3, and 5 h after amodiaquine administration, and serum drug levels, circulating biomarkers, and hepatic candidate transcripts were quantified. These serum and hepatic markers were selected based on prior studies showing that the IDR-associated drugs nevirapine (Jee et al. 2024) and clozapine (Sernoskie et al. 2022) induced early increases in several of the same readouts. This approach allowed assessment of whether amodiaquine elicited a comparable early response and helped identify the optimal sampling time for the subsequent comparative study.

**Fig. 1.**
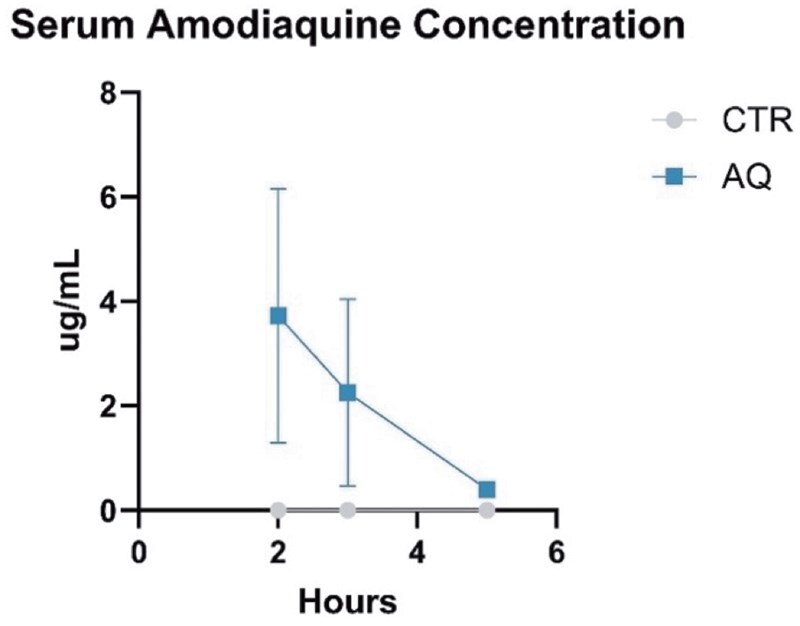
Serum amodiaquine exposure over time. Mice received amodiaquine (AQ) or vehicle control (CTR), and serum amodiaquine concentrations were quantified by LC–MS/MS at 2, 3, and 5 h post-administration. Points show group means with error bars indicating variability (SD).

**Fig. 2.**
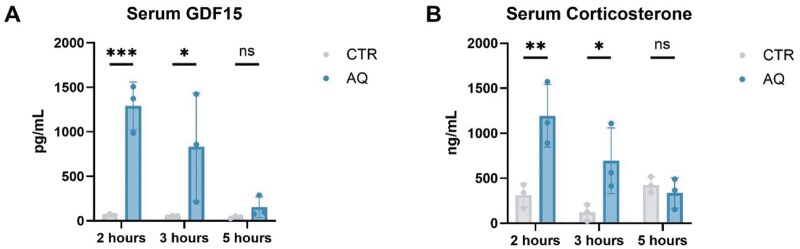
Time-dependent changes in circulating GDF15 and corticosterone following amodiaquine administration. Mice received amodiaquine (AQ) or vehicle control (CTR) and serum was collected at 2, 3, and 5 h post-administration. (A) Serum GDF15 concentrations were measured by ELISA. (B) Serum corticosterone concentrations were measured by ELISA. Data are presented as mean ± SD, n = 3 per group. Statistical comparisons were made using 2-way ANOVA; Statistical significance between treated and time-matched control groups is indicated: Ns = not significant, *P ≤ 0.05, **P ≤ 0.01, ***P ≤ 0.001.

Liquid chromatography–tandem mass spectrometry (LC–MS/MS) confirmed substantial systemic exposure to amodiaquine at early timepoints, with serum concentrations highest at 2 to 3 h and declining markedly by 5 h (Fig. 1). Consistent with this exposure window, serum GDF15 was strongly elevated at 2 h and remained significantly increased at 3 h but returned toward control levels by 5 h (Fig. 2A). A similar pattern was observed for serum corticosterone, which was significantly elevated at 2 and 3 h, with no significant increase at 5 h (Fig. 2B). Together, these findings indicate that both circulating readouts changed early after dosing and were attenuated by 5 h, with the largest dynamic range captured within the first 3 h after dosing.

**Fig. 3.**
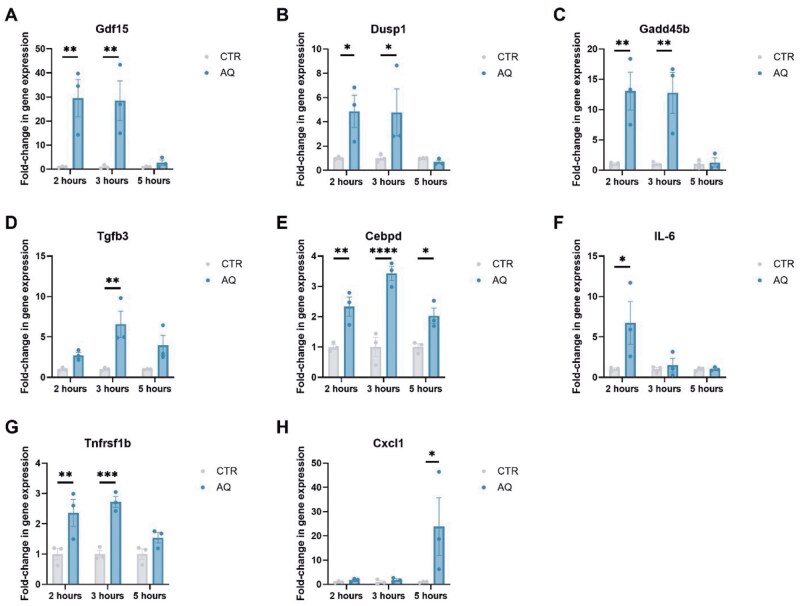
Time-course of hepatic transcriptional responses to amodiaquine measured by RT–qPCR. Mouse livers were collected at 2, 3, and 5 h after amodiaquine treatment or time-matched vehicle control, and selected gene targets were quantified by RT–qPCR. Relative mRNA expression (fold change vs time-matched control after normalization to reference gene) is shown for Gdf15 (A), Dusp1 (B), Gadd45b (C), Cebpd (D), Tgfb3 (E), Il6 (F), Tnfrsf1b (G), and Cxcl1 (H). Data are presented as mean ± SEM, n = 3 per group. Statistical comparisons were made using 2-way ANOVA; ns = not significant, *P ≤ 0.05, **P ≤ 0.01, ***P ≤ 0.001, ****P ≤ 0.0001.

Hepatic RT–qPCR profiling showed similar temporal kinetics. *Gdf15*, *Dusp1*, and *Gadd45b* were robustly induced at 2 h and 3 h, followed by a return toward baseline by 5 h (Fig. 3A–C). In contrast, *Cebpd* remained significantly elevated across all timepoints (Fig. 3D). Among immune-associated targets, *Tgfb3* appeared to peak at 3 h, whereas Tnfrsf1b was elevated at 2 to 3 h (Fig. 3G). *Cxcl1* showed a delayed increase that became apparent at 5 h (Fig. 3H), suggesting that a subset of transcriptional changes emerged later than the early stress-gene response. Notably, *Il6* was significantly increased at 2 h (Fig. 3F). Because this increase was not reproduced consistently across later experiments, it is described here as an observation limited to this time-course study.

Overall, this time-course experiment demonstrates that amodiaquine induces an early response characterized by rapid increases in both circulating biomarkers and hepatic stress-response transcripts, with attenuation of many of these signals by 5 h. These kinetics indicate that sampling within 2 to 3 h is optimal for capturing early responses and therefore supported the selection of the 2 h timepoint for subsequent comparative experiments.

### In vivo comparison reveals differential induction of hepatic stress genes and circulating biomarkers across IDR-associated drugs

**Fig. 4.**
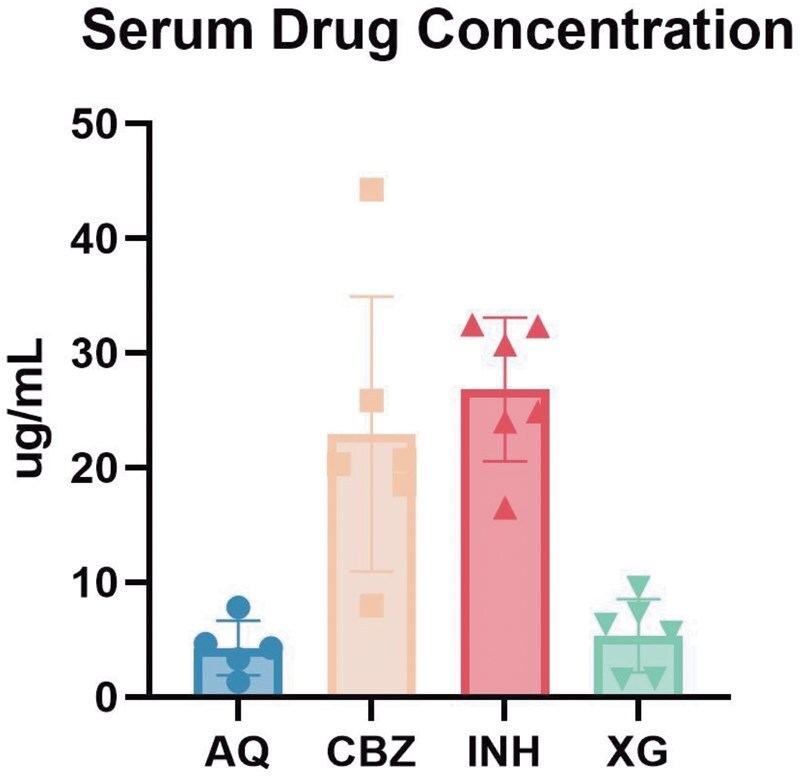
Serum drug concentrations 2 h post-administration. Serum concentrations of amodiaquine (AQ), carbamazepine (CBZ), isoniazid (INH), and ximelagatran (XG) were quantified by LC–MS/MS at the experimental sampling timepoint (2 h post-administration). Each symbol represents an individual mouse (n = 6 per group), and bars show group summary statistics (mean ± SD).

To assess whether the identified markers were generalizable across other IDR-associated drugs, a comparative in vivo experiment was performed in which mice received carbamazepine, isoniazid, amodiaquine, ximelagatran, or vehicle and were sampled 2 h post-dose. Intraperitoneal administration was used to minimize the variability in drug exposure that we had observed with the oral route (data not shown). LC–MS/MS confirmed detectable serum concentrations for all administered compounds at 2 h post-dose, with values broadly consistent with therapeutically relevant exposure (Eriksson et al. 2003; Babalik et al. 2011; Metushi et al. 2015; Greenberg et al. 2016) (Fig. 4).

**Fig. 5.**
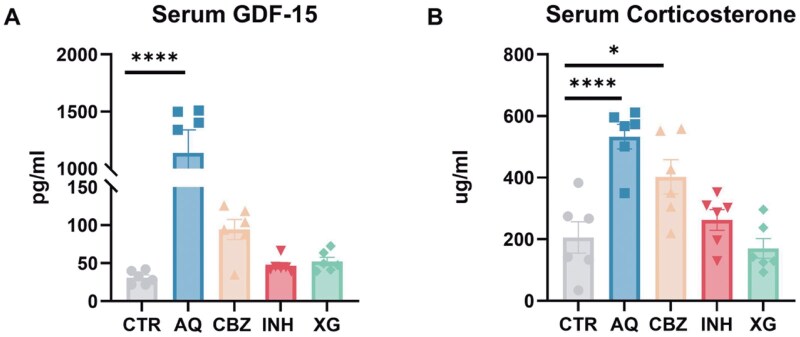
Circulating biomarker levels following drug administration. Mice received vehicle control (CTR) or a single i.p. dose of amodiaquine (AQ), carbamazepine (CBZ), isoniazid (INH), or ximelagatran (XG) (n = 6 per group), and serum was collected at 2 h post-administration. (A) Serum GDF-15 concentrations were measured by ELISA. (B) Serum corticosterone concentrations were measured by ELISA. Data are presented as mean ± SD. Statistical significance was determined by 1-way ANOVA with multiple comparisons; *P ≤ 0.05, *** *P* ≤ 0.0001.

The circulating biomarker responses were drug dependent. Serum GDF15 increased robustly following amodiaquine, whereas the change with carbamazepine was not statistically significant, and there was no change with isoniazid or ximelagatran (Fig. 5A). Serum corticosterone was significantly elevated after amodiaquine and carbamazepine, whereas with isoniazid and ximelagatran, it remained close to control levels (Fig. 5B).

**Fig. 6.**
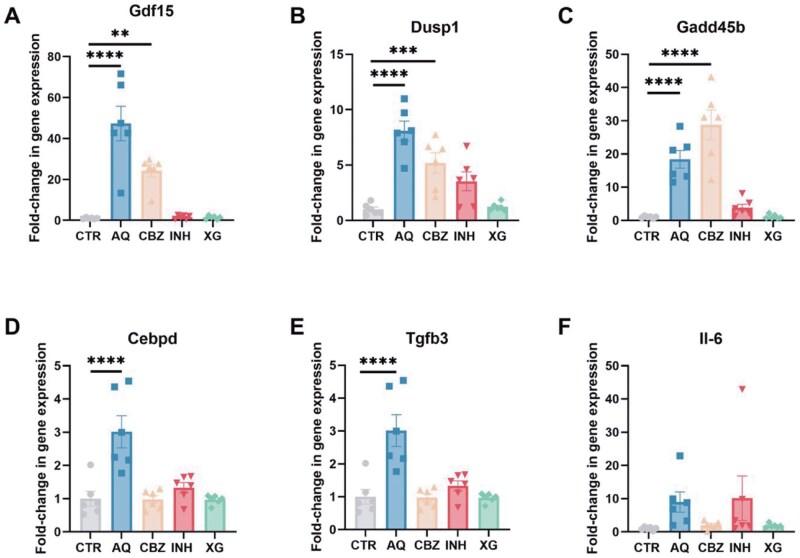
Comparative hepatic RT–qPCR analysis of selected genes following drug administration. Mouse livers were collected 2 h after i.p. dosing with carbamazepine (CBZ), amodiaquine (AQ), isoniazid (INH), ximelagatran (XG), or vehicle control (CTR). Relative mRNA expression (fold change vs CTR after normalization to reference gene) was quantified by RT–qPCR for Gdf15 (A), Dusp1 (B), Gadd45b (C), Cebpd (D), Tgfb3 (E), and Il6 (F). Data are shown as mean ± SEM, n = 6 per group. Statistical significance was determined by 1-way ANOVA with multiple comparisons; *P ≤ 0.05, ** *P* ≤ 0.001, *** *P* ≤ 0.0001.

In the liver, carbamazepine produced a smaller but significant induction of *Gdf15*, *Dusp1*, and *Gadd45b* compared with amodiaquine. The apparent discrepancy between hepatic *Gdf15* mRNA induction and serum GDF15 protein levels may reflect differences in the temporal kinetics of transcriptional activation and subsequent protein accumulation or release into the circulation. An independent follow-up study at 3 h was subsequently performed to further confirm the carbamazepine effects, as described in the next section. In contrast, isoniazid and ximelagatran showed limited early induction of these targets under the conditions tested. *Il6* did not increase across treatment groups, suggesting that, at this early timepoint, the measured response was dominated by the selected stress-response markers rather than by direct immune activation (Fig. 6).

The weak response observed for ximelagatran was consistent with expectations, whereas the minimal response observed with isoniazid was unexpected. Because isoniazid is bioactivated in the liver, the lack of induction in this targeted panel prompted a broader transcriptomic comparison of isoniazid and carbamazepine to determine whether the 2 drugs elicited fundamentally different early hepatic responses.

### Hepatic transcriptomic profiling of isoniazid reveals divergent early responses from carbamazepine and isoniazid

To investigate the unexpected lack of response to isoniazid in the targeted qPCR analysis, early hepatic transcriptional responses to carbamazepine and isoniazid were examined by RNA-seq. Mouse liver was collected 3 h after i.p. administration of carbamazepine (100 mg/kg) or isoniazid (100 mg/kg) and compared with time-matched vehicle controls (n = 4 per group). Global structure of the datasets was evaluated by principal component analysis (PCA), followed by visualization of differentially expressed genes (DEGs) using hierarchical clustering heatmaps and volcano plots.

**Fig. 7.**
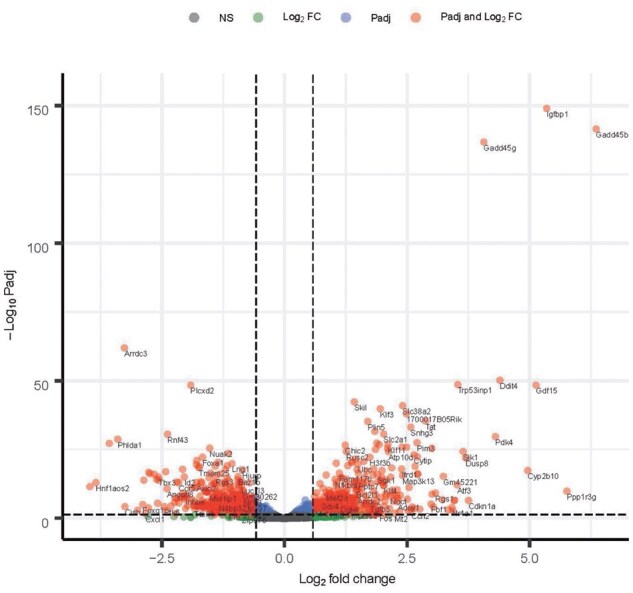
Differential gene expression in mouse liver after carbamazepine treatment. Volcano plot of differential gene expression in mouse liver between control and carbamazepine-treated samples. The *x*-axis shows log_2_ fold change and the *y*-axis shows − log_10_(Padj). Dashed vertical lines mark the fold-change cutoff (|log_2_FC| = 0.58; 1.5-fold), and the dashed horizontal line marks the significance cutoff (Padj = 0.05). Genes are colored by whether they meet the fold-change threshold, the Padj threshold, or both; selected genes are annotated.

In the carbamazepine cohort, PCA of filtered genes showed a clear separation between control and carbamazepine-treated liver samples along the major axis of variance (PC1 = 49%, PC2 = 16%; Fig. S1), indicating a robust treatment-associated shift in the hepatic transcriptome. Hierarchical clustering of DEGs revealed tight grouping of carbamazepine-treated samples (Fig. S3). Differential expression analysis identified 1,351 DEGs meeting the thresholds used in the figure (Padj < 0.05 and |log_2_FC| > 0.58), including 572 upregulated and 779 downregulated transcripts in carbamazepine-treated liver relative to control (Fig. 7).

Carbamazepine-induced DEGs were then compared with a previously published nevirapine liver RNA-seq dataset (Jee et al. 2024). This analysis identified 51 genes commonly upregulated and 20 genes commonly downregulated by both carbamazepine and nevirapine even though the pharmacology of these 2 drugs are quite different (Table S2). Shared induced genes included stress- and immediate early response genes such as *Gadd45b*, *Ddit4*, *Fos*, *Jun*, *Dusp1*, *Cebpd*, and *Gdf15*, together with metabolic and remodeling-associated genes including *Cyp2b10*, *Pck1*, *Angptl4*, *Ppargc1a*, and *Ppargc1b*. Commonly downregulated genes included *Srebf1*, *Cxcl1*, *Cd180*, and *Lcp2*. These concordant changes define a set of early transcriptional responses shared by carbamazepine and nevirapine, which appear to involve oxidative and cellular stress signaling, lipid and fatty acid metabolic regulation, and xenobiotic metabolism.

**Fig. 8.**
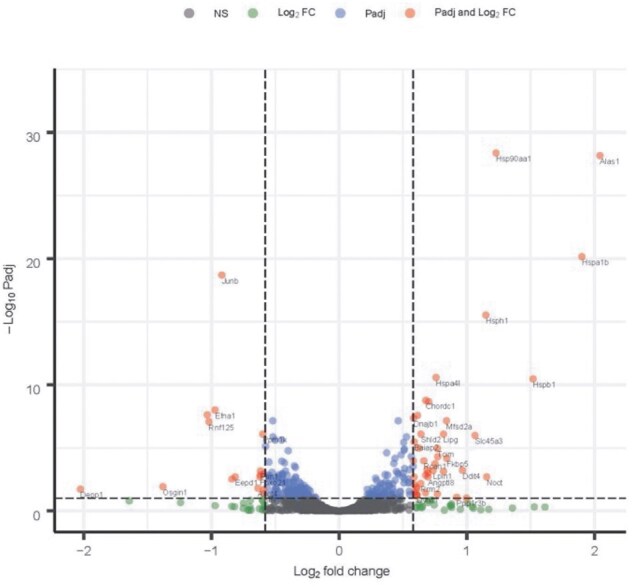
Differential gene expression in mouse liver after isoniazid treatment. Volcano plot of differential gene expression in mouse liver comparing isoniazid-treated vs control samples. The *x*-axis shows log_2_ fold change (treatment vs control) and the *y*-axis shows − log_10_(Padj). Dashed vertical lines indicate the fold-change cutoff (|log_2_FC| = 0.58; 1.5-fold), and the dashed horizontal line indicates the Padj cutoff (0.1). Points are colored by significance category (fold change, Padj, or both), and selected genes are annotated.

In contrast, the isoniazid cohort showed a more moderate separation of treated and control liver samples in PCA space (PC1 = 30%, PC2 = 24%; Fig. S2). Although separation between groups was less pronounced than that observed with carbamazepine, hierarchical clustering still showed treatment-associated structure with isoniazid-treated samples exhibiting an expression shift relative to controls (Fig. S4). Using the filtering criteria (Padj < 0.1, |log_2_FC| > 0.58, and expression filter of ≥3 FPKM in at least 2 samples), differential expression analysis identified 62 DEGs, including 44 upregulated and 18 downregulated genes, in isoniazid-treated liver compared with control (Fig. 8). Notably, the most induced transcripts included multiple heat shock and chaperone genes involved in proteostasis, including *Hspa1b*, *Hspb1*, *Hsp90aa1*, and *Hsph1*. Thus, consistent with the targeted qPCR findings, isoniazid elicited a much narrower early DEG signature than carbamazepine and appeared to engage a distinct set of cellular stress-response pathways.

**Fig. 9.**
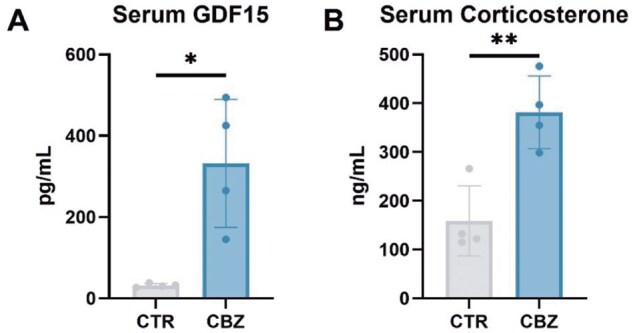
Effect of carbamazepine on serum GDF15 and corticosterone at 3 h post-dose.Mice were treated with carbamazepine (100 mg/kg, i.p.) or vehicle control and serum was collected 3 h later. Serum concentrations of (A) GDF15 and (B) corticosterone were measured by ELISA. Data are presented as mean ± SD, with individual animals shown (n = 4 per group). Statistical significance was determined by unpaired 2-tailed *t*-test. *P < 0.05, **P < 0.01 versus control.

**Fig. 10.**
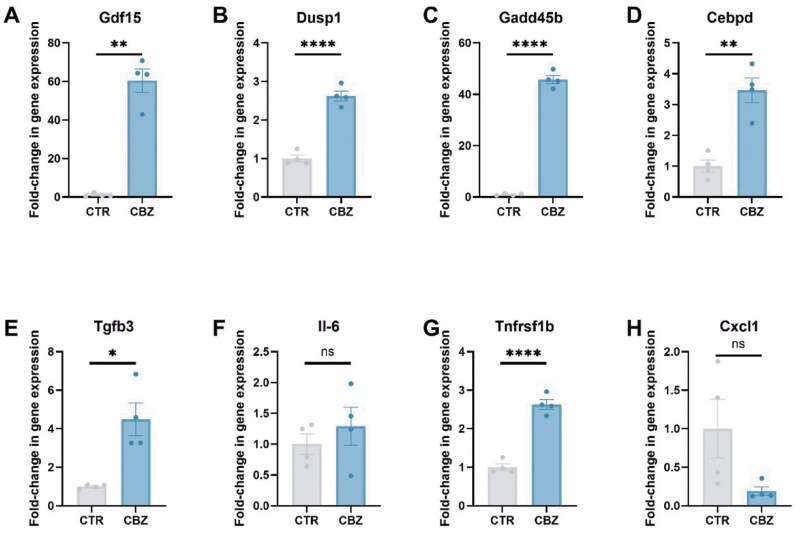
Effect of carbamazepine on hepatic gene expression at 3 h post-dose. Mice were treated with carbamazepine (100 mg/kg, i.p.) or vehicle control and liver tissue was collected 3 h later. Hepatic mRNA expression of (A) *Gdf15*, (B) *Dusp1*, (C) *Gadd45b*, (D) *Cebpd*, (E) *Tgfb3*, (F) *Il6*, (G) *Tnfrsf1b*, and (H) *Cxcl1* was measured by RT-qPCR. Gene expression was normalized to cyclophilin and is shown as fold change relative to control. Data are presented as mean ± SEM, with individual animals shown (n = 4 per group). Statistical significance was determined by unpaired 2-tailed *t*-test. *P < 0.05, **P < 0.01, ****P < 0.0001 versus control; ns, not significant.

In the comparative 2 h experiment described above, carbamazepine showed a nonsignificant upward trend in serum GDF15 and hepatic *Cebpd* mRNA expression. However, RNA-seq analysis showed that both *Gdf15* and *Cebpd* were significantly increased. To confirm these findings, an independent follow-up experiment was performed in mice treated with the same dose of carbamazepine, and liver samples were collected 3 h after administration for real-time quantitative polymerase chain reaction (RT-qPCR) analysis. Consistent with the RNA-seq results, both markers were significantly increased at 3 h (Figs 9 and 10). These findings indicate dynamics of early drug responses, highlighting the importance of including multiple timepoints in future experiments.

## Discussion

The development of biomarkers that predict the risk that a drug candidate would cause a significant incidence of iDILI would have a major effect on drug development. We developed an animal model of iDILI by using PD-1 KO mice and anti-CTLA-4 to impair immune tolerance. It is similar to human iDILI because it is delayed in onset and mediated by CD8+ T cells (Mak and Uetrecht 2015a). The injury is mild because it depends on random weak interactions with MHC and T cell receptors, but it has the potential to predict iDILI risk. However, it is not very practical for screening drug candidates because it is delayed by 3 wk or more, and it requires a large amount of drug to treat animals for that length of time. In vitro assays are very attractive, but they lack the complexity of an intact immune system. Therefore, we have chosen to study the initial response to drugs in mice as potential biomarkers of iDILI risk. Later, we will determine if similar changes can be reproduced in vitro.

An essential step in the induction of an immune-mediated idiosyncratic drug reaction such as iDILI is the activation of APCs such as macrophages. This represents signal 2, which is required for activation of an adaptive immune response (Uetrecht 2025). This activation is likely mediated by molecules released by hepatocytes in response to cell stress. We have found that incubation of THP-1-derived macrophages with the supernatant from incubation of hepatocytes with drugs such as nevirapine and carbamazepine leads to activation of inflammasomes and the release of IL-1β (Kato and Uetrecht 2017; Kato et al. 2019). Consistent with previous results with clozapine and nevirapine, in the present study, carbamazepine and amodiaquine produced a very rapid increase in hepatic gene transcription consistent with a stress/innate immune response. Together, these findings support the use of early stress- and inflammation-responsive transcripts as potential biomarkers of the hepatic events that precede APC activation. GDF-15 is widely recognized as a marker of cellular stress and tissue injury (Schwarz et al. 2023), whereas DUSP1 (Smallie et al. 2015)and GADD45B (Ma et al. 2025) are rapidly inducible stress-response genes involved in MAPK regulation, inflammatory signaling, and cellular adaptation to injury. In addition, CEBPD is a transcription factor involved in immune and inflammatory gene regulation and macrophage activation/differentiation (Spek et al. 2021), making these markers biologically relevant to the proposed link between hepatocyte stress and downstream innate immune activation. There was also an increase in serum corticosterone and GDF-15. These changes occurred at nontoxic doses, and in most cases, close to the therapeutic range in humans. It is likely that such responses represent biomarkers that could be used to screen drug candidates for iDILI risk during drug candidate selection. Of course, they would not be expected to predict which patients would develop iDILI. This is analogous to findings in a study of changes that were produced in hepatocytes in vitro (Bergen et al. 2025); however, those results are impossible to fully evaluate because detailed data were not presented.

In contrast, the changes that we believe to be the most relevant biomarkers of iDILI risk did not occur with isoniazid and ximelagatran treatment. It is clear that the iDILI associated with these drugs is immune-mediated. Ximelagatran iDILI is associated with a specific HLA haplotype, and it also appears to be a contact sensitizer (Kindmark et al. 2008). Although isoniazid iDILI has not been linked to a specific HLA haplotype, it is associated with anti-drug antibodies, and the histology is characteristic of an immune-mediated reaction (Metushi et al. 2014). As mentioned earlier, the lack of response to ximelagatran was not unexpected. In fact, ximelagatran was chosen because we expected the response to be different. Unlike the other drugs studied, ximelagatran is not metabolized via cytochrome P450 enzymes or myeloperoxidase, and it does not appear to form reactive metabolites (Kenne et al. 2008). However, as noted, it has been reported to directly activate macrophages (Edling et al. 2008).

What is surprising was the limited response to isoniazid. Heat shock proteins represent DAMPs and could activate APCs. For example, the supernatant from the incubation of hepatocytes with gefitinib and other drugs activated THP-1-derived macrophages, and this supernatant contained heat shock proteins which were postulated to be responsible for this activation (Kato et al. 2020). It is also possible that the response in mice is different from that in humans; however, we found that isoniazid also produced an immune-mediated liver injury in PD-1^−/−^ mice (Mak and Uetrecht 2015b). Isoniazid is known to undergo bioactivation in the liver and has been shown to covalent bind to hepatic proteins in rodents and to human liver microsomes in vitro (Metushi et al. 2012). In addition, isoniazid is also oxidized by neutrophils, presumably by myeloperoxidase, so it should also be oxidized by macrophages, although unlike clozapine and amodiaquine it does not appear to cause agranulocytosis (Hofstra et al. 1992). Isoniazid can also cause other immune-mediated adverse reactions such as a lupus-like syndrome and drug fever, which is typical of drugs oxidized to reactive metabolites by myeloperoxidase, and it is consistent with direct activation of macrophages (Uetrecht 2026). In addition, hydrazines such as isoniazid are very good nucleophiles and may directly activate macrophages by interaction with signaling molecules on macrophages (Li and Uetrecht 2009). A preliminary experiment to determine if isoniazid can activate inflammasomes in THP-1-derived macrophages was negative (results not shown); however, inflammasome activation is only one measure of macrophage activation, and THP-1-derived macrophages do not have the full range of responses of normal macrophages (Tedesco et al. 2018). It is also possible that drugs could express or release molecules that activate APCs without an increase in expression of mRNAs. Furthermore, a combination of the production of DAMPs and direct activation of APCs may contribute to the ability of a drug to cause iDILI.

In summary, most iDILI is immune-mediated, and activation of APCs to produce signal 2 is necessary but not sufficient to produce an adaptive immune response that in some patients leads to iDILI. We believe that upregulation of specific hepatic mRNAs such as *GDF-15*, *Dusp-1*, *Gadd45b*, and *Cebpd* represent biomarkers of iDILI risk and would improve drug safety. However, without measures of direct APC activation or other mechanisms of APC activation, such biomarkers would be incomplete.

## Supplementary Material

kfag087_Supplementary_Data
