# Supplementary material for: Hepatic responses as potential biomarkers of idiosyncratic drug reaction risk
Source: Toxicol Sci. 2026 Jul 29;209(8):kfag087. doi: 10.1093/toxsci/kfag087 (PMC13452458; doi:10.1093/toxsci/kfag087)
Supplement: Supplementary file 1 [file kfag087_supplementary_data.pdf]

Hepatic Responses as Potential Biomarkers of Idiosyncratic Drug Reaction Risk

Jiamin Liao[1], Xiaotian Lyu[1], Kristy Yang[2], Lucia Chen[1], Jack Uetrecht[1,2]

[1]Department of Pharmacology & Toxicology, Temerty Faculty of Medicine, University of Toronto

[2]Department of Pharmaceutical Sciences, Leslie Dan Faculty of Pharmacy, University of Toronto

Supplementary Material

Supplemental tables

Supplemental Table 1. Primer sequences used for RT-qPCR

Gene symbol	RefSeq	Forward primer	Reverse primer
Gdf15	NM_011819.4	CGC CCT GGC AAT GCC TGA ACA ACG AC	GCA CGC GGT AGG CTT CGG GGA GAC C
Dusp1	NM_013642.3	AAC AGG GCA GAA GAG AAA GG	TCA TCG GGA ATG GTT AAT ACT G
Gadd45b	NM_008655.1	CTG CCT CCT GGT CAC GAA	TTG CCT CTG CTC TCT TCA CA
Cebpd	NM_007679.4	TCG ACT TCA GCG CCT ACA TTG ACT	CCG CTT TGT GGT TGC TGT TGA AGA
Tgfb3	NM_009368.3	TGG CTG TTG AGG AGA GAG TCC	TGT GGG TTG TGG TGA TCC TT
Il6	NM_031168.2	GGG ACT GAT GCT GGT GAC AA	TGC CAT TGC ACA ACT CTT TTC
Tnfrsf1b	NM_011610.3	TCT GTC ATC TCG GAA GCC CA	GAG CCC TAA GCT GGT CTA AGC
Cxcl1	NM_008176.3	GGT GTC CCC AAG TAA CGG AG	TTG TCA GAA GCC AGC GTT CA
Cyclophilin	NM_011149.2	GGA GAT GGC ACA GGA GGA A	GCC CGT AGT GCT TCA GCT T

Supplementary Table 2. Common hepatic gene expression changes shared by carbamazepine and nevirapine in mouse liver RNA-seq

Gene	Carbamazepine	Nevirapine
Gadd45b	Up	Up
Ppp1r3g	Up	Up
Igfbp1	Up	Up
Gdf15	Up	Up
Cyp2b10	Up	Up
Ddit4	Up	Up
Mfsd2a	Up	Up
Rgs16	Up	Up
Jun	Up	Up
Pim3	Up	Up
Synj2	Up	Up
Btg2	Up	Up
Ccn2	Up	Up
Fam107a	Up	Up
Slc38a2	Up	Up
Ifrd1	Up	Up
Adcy1	Up	Up
Per1	Up	Up
Mt1	Up	Up
Slc10a2	Up	Up
Ptp4a1	Up	Up
Dusp1	Up	Up
Tnfrsf1b	Up	Up
Apbb3	Up	Up
Bcl2l1	Up	Up

Epha2	Up	Up
Skil	Up	Up
Klf3	Up	Up
Glt1d1	Up	Up
Ackr2	Up	Up
Angptl4	Up	Up
Tmem26	Up	Up
Rusc2	Up	Up
Pck1	Up	Up
Fos	Up	Up
Slc25a51	Up	Up
Acta2	Up	Up
Actg1	Up	Up
Pla2g12a	Up	Up
Cep85l	Up	Up
Olfml3	Up	Up
Lama2	Up	Up
Slc16a6	Up	Up
Ppargc1a	Up	Up
Ppargc1b	Up	Up
Ldlr	Up	Up
Slc7a2	Up	Up
Hspa2	Up	Up
Cebpd	Up	Up
Map3k6	Up	Up
Rhobtb1	Up	Up
Fgfr3	Down	Down
Inhbe	Down	Down

Rab3il1	Down	Down
Rnf43	Down	Down
Srebf1	Down	Down
Cd180	Down	Down
Tiam2	Down	Down
Mid1ip1	Down	Down
Ptk6	Down	Down
Lcp2	Down	Down
Fzd7	Down	Down
Wnt4	Down	Down
Rgs19	Down	Down
Dbp	Down	Down
Cxcl1	Down	Down
Cish	Down	Down
Phlda1	Down	Down
Nrep	Down	Down
Adap1	Down	Down
Cbx2	Down	Down

Supplemental figures

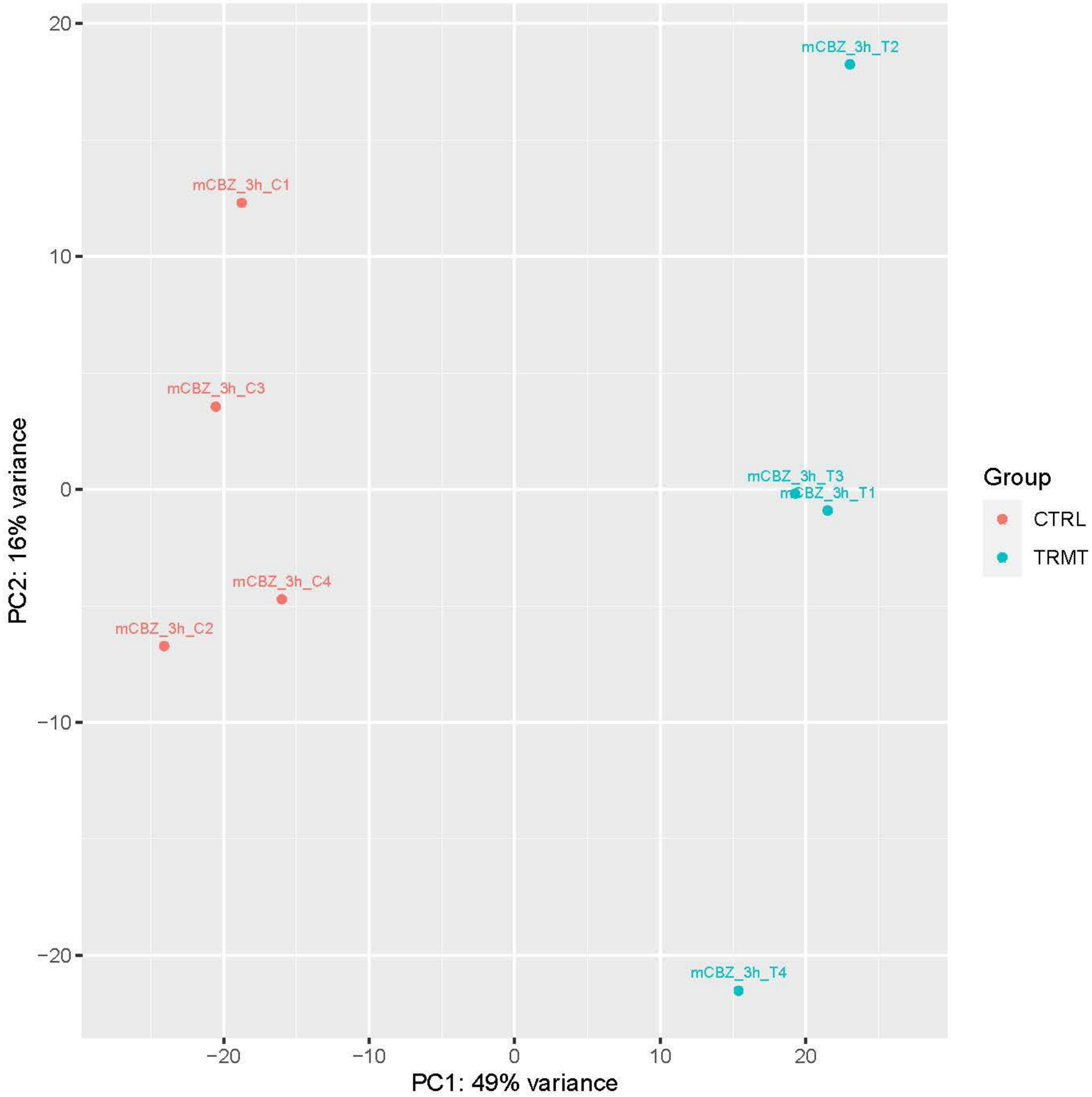

Supplementary Figure 1. Principal component analysis (PCA) of mouse liver RNA-seq samples based on filtered gene expression, showing separation of control (CTRL) and carbamazepine-treated (TRMT) groups along PC1 (49% variance) and PC2 (16% variance). Each point represents one sample (n = 4 per group).

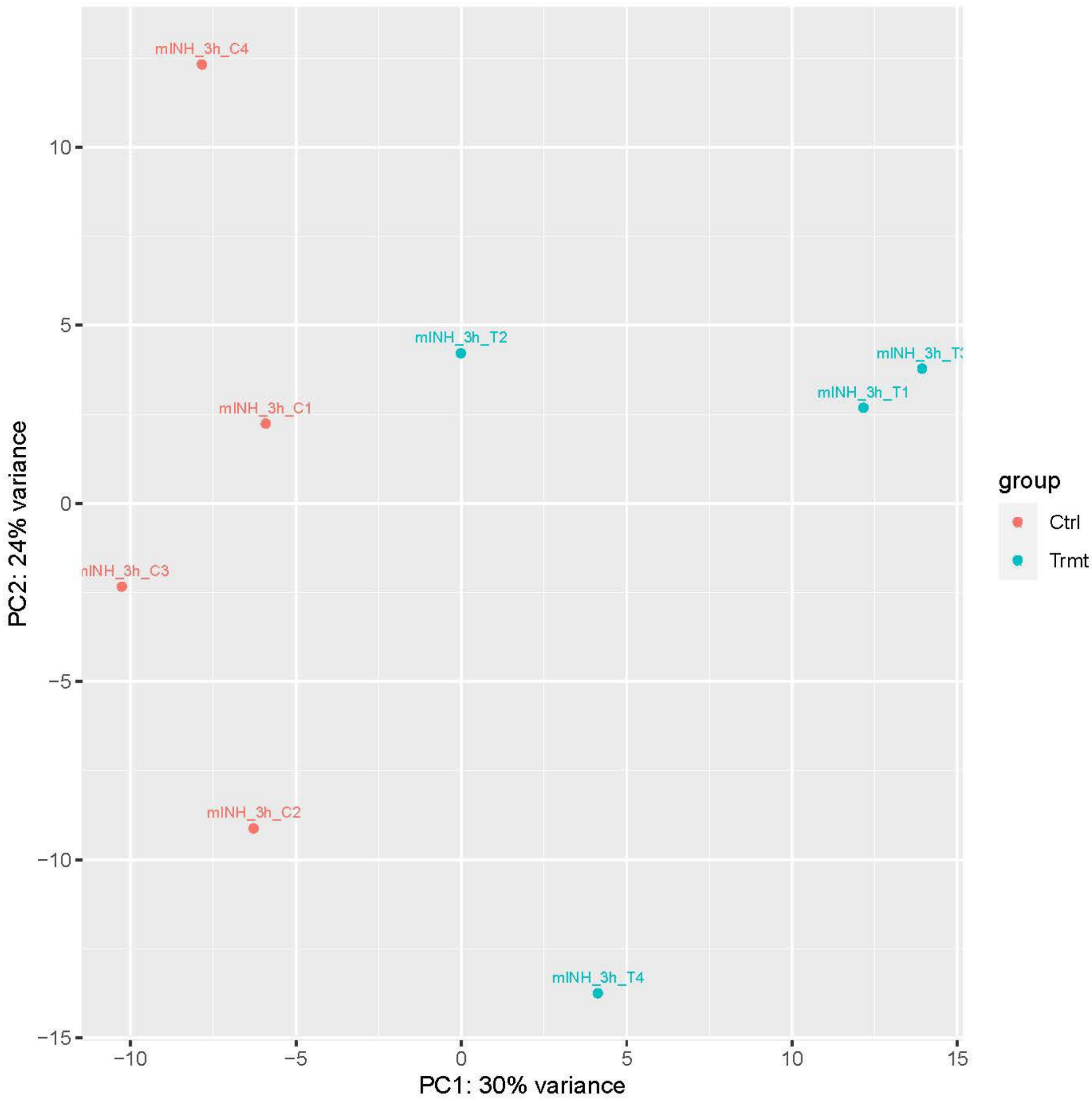

Supplementary Figure 2. Principal component analysis (PCA) of mouse liver RNA-seq samples based on filtered gene expression, showing separation of control (Ctrl) and isoniazid-treated (Trmt) samples along PC1 (30% variance) and PC2 (24% variance). Each point represents one sample (n = 4 per group; sample IDs labelled).

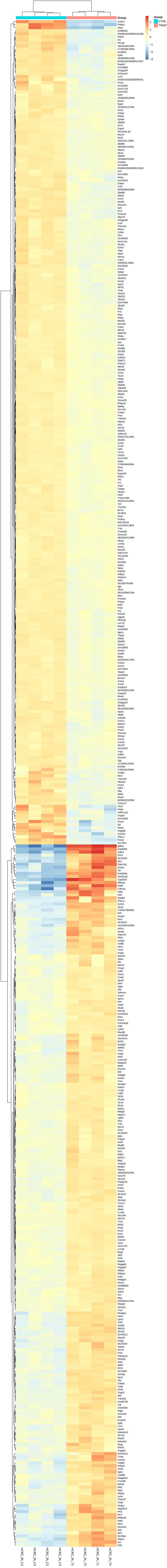
Group
CTRL
TRMT

Supplementary Figure 3. Heatmap of differentially expressed genes in mouse liver following carbamazepine treatment. Mouse livers were collected 3 h after i.p. administration of carbamazepine (100 mg/kg) and compared with time-matched vehicle controls (n = 4 per group). The heatmap displays genes identified as differentially expressed (Padj < 0.05 and $|log_2FC| > 0.58$). Columns represent individual samples and rows represent genes; values are shown as row-scaled relative expression. Both genes and samples were hierarchically clustered. The top annotation indicates treatment group (control/CTRL vs carbamazepine/TRMT).

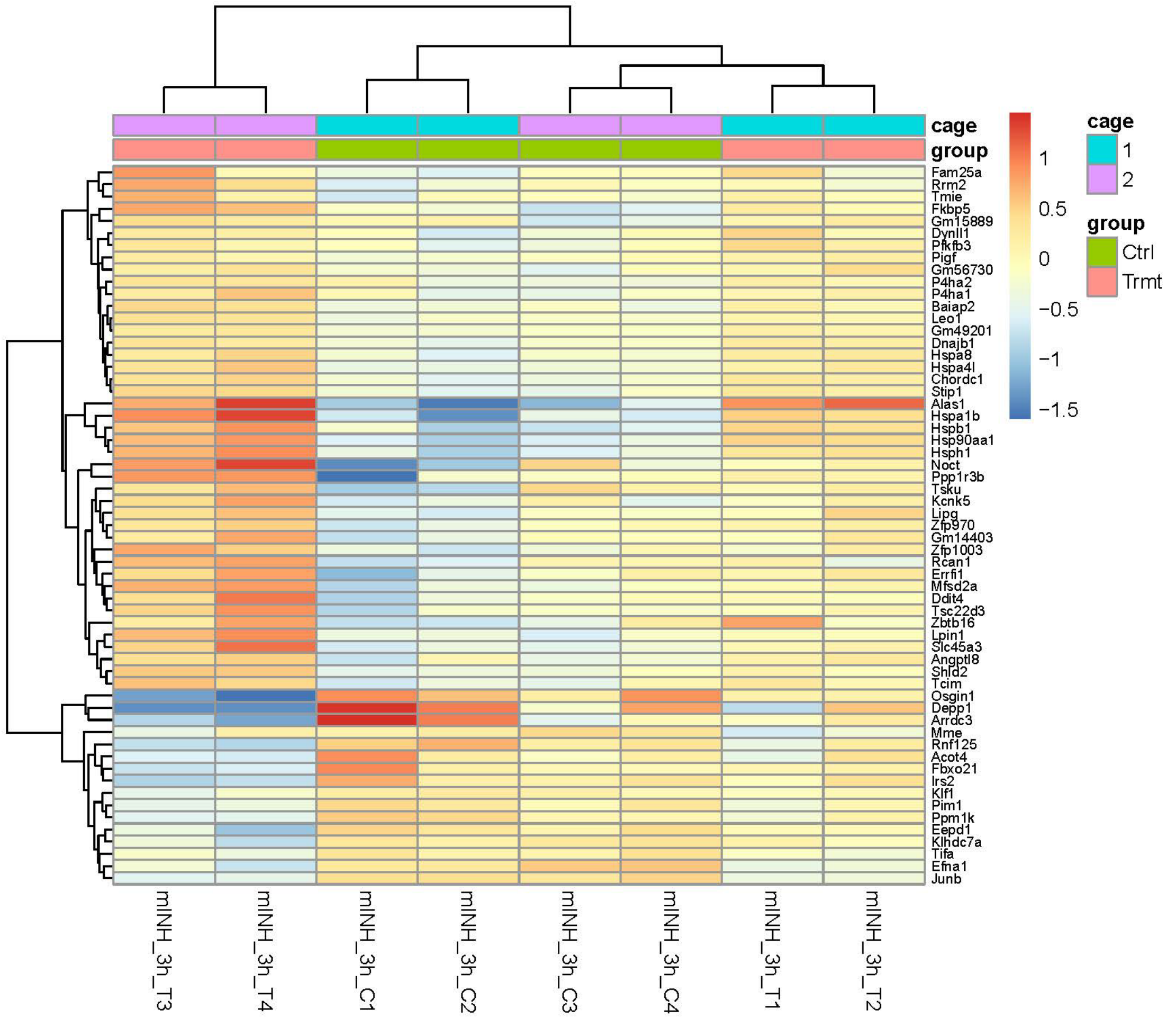

Supplementary Figure 4. Heatmap of differentially expressed genes in mouse liver between isoniazid-treated (Trmt) and control (Ctrl) groups. Genes shown meet the differential-expression and expression filters ($|log2FC| > 0.58$ and Padj < 0.1; minimum expression ⩾3 FPKM in at least two samples). Rows (genes) and columns (samples) are hierarchically clustered. Colors indicate row-scaled relative expression, and the top annotations indicate cage and treatment group.
